# Fus knockdown inhibits the profibrogenic effect of cardiac fibroblasts induced by angiotensin II through targeting Pax3 thereby regulating TGF-β1/Smad pathway

**DOI:** 10.1080/21655979.2021.1918522

**Published:** 2021-04-25

**Authors:** Guoqiang Wang, Hong Wu, Peng Liang, Xiaojiao He, Dong Liu

**Affiliations:** a Department of Cardiology, Chongqing Kanghua Zhonglian Cardiovascular Hospital, Chongqing, China; b Department of Obstetrics and Gynecology, The People’s Hospital, Chongqing, China; c Department of Cardiology, The People’s Hospital, Chongqing, China

**Keywords:** Angiotensin II, atrial fibrillation, fibrosis, fused in sarcoma, transforming growth factor

## Abstract

The Angiotensin II/transforming growth factor-β1 (AngII/TGF-β1) signal axis is an important regulatory pathway for atrial fibrosis, which can contribute to atrial fibrillation (AF). Fused in sarcoma (FUS) was recently found to regulate cardiac diseases. This study aimed to investigate whether FUS could regulate AngII induced fibrosis and uncover the possible mechanisms. The expression of FUS in AF patients and AngII-induced cardiac fibroblasts was measured by RT-qPCR and western blot assays. Fus was silenced in cells using short hairpin RNA (shRNA), then cell proliferation, migration, collagen synthesis and TGF-β1/Smad signaling were detected by CCK-8, wound healing and western blot assays, respectively. The possible target for Fus was predicted by searching Starbase database and verified by RNA-binding protein immunoprecipitation (RIP) and RNA pull down. Cells were overexpressed with Pax3 in the presence of Fus silence and AngII stimulation, then the above cellular processes were further evaluated. Results showed that FUS was upregulated in AF patients and AngII-induced cardiac fibroblasts. Fus knockdown inhibited AngII-enhanced cell proliferation, migration, collagen synthesis and TGF-β1/Smad signaling activation. Furthermore, Fus functions as an RNA-binding protein to bind to Pax3 mRNA and positively regulate its expression. Further studies demonstrated that Pax3 overexpression canceled the above effects of Fus knockdown on cell proliferation, migration, collagen synthesis, and TGF-β1/Smad signaling activation in AngII-induced cells. In conclusion, Fus could target Pax3 to increase the pro-fibrotic effect of AngII in cardiac fibroblasts via activating TGF-β1/Smad signaling. Knockdown of Fus/Pax3 axis may provide a potential therapy for relieving AF.

## Graphical Abstract

**Figure:**
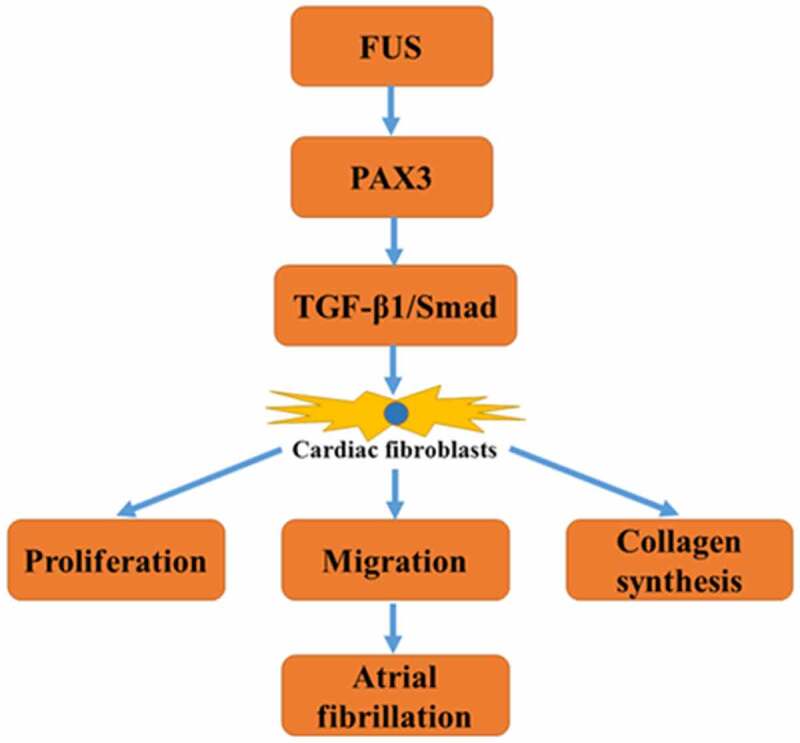

## Introduction

Atrial fibrillation (AF) is an arrhythmia characterized by rapid, asynchronous atrial activation and ineffective atrial contractions. AF can easily result in complications such as stroke and limb embolism, which significantly increase the mortality and disability rate of cardiovascular disease [1]. At present, the treatment of AF still has many limitations, including low effectiveness, postoperative malignant arrhythmia, and high recurrence rate, the fundamental reason of which is that the mechanism of AF is not thoroughly studied [2]. Therefore, an in-depth study of the underlying molecular mechanisms of AF is a prerequisite for improving clinical efficacy.

In the past, research on AF has mainly focused on electrophysiology. Increasing studies have shown that changes in the content and configuration of myocardial collagen fibers are the cause of cardiac remodeling, and atrial fibrosis is considered to be the common pathological basis for many causes of AF [3–5]. As a result, to explore pathological mechanism of atrial fibrosis and find effective intervention targets against atrial fibrosis are the current focus of the current research on AF. The formation of atrial fibrosis is affected by multiple signaling pathways, among which, the Angiotensin II/transforming growth factor-β1 (AngII/TGF-β1) signal axis is considered to be the most important regulatory pathway for atrial fibrosis [6–8]. For instance, the inhibition of TGF-β1/Smad2/3 pathway can attenuate AngII-induced atrial fibrosis and vulnerability to AF [9].

Fused in sarcoma (FUS) is widely expressed multifunctional protein and participates in multiple biological processes, such as DNA repair, gene transcription, oxidative stress, and mitochondrial damage [10]. Recent studies have confirmed that FUS is involved in multiple cardiac diseases. For example, lncRNA KCNQ1OT1 was reported to contribute to cardiomyocyte apoptosis by targeting FUS in heart failure [11]. In cardiac hypertrophy, FUS acts as an mRNA stabilizer and can be recruited by lncRNA CTBP1-AS2 to stabilize toll-like receptor (TLR)4 [12]. However, whether FUS could regulate cardiac fibrosis has not been illustrated.

After searching Starbase database, we found that FUS can function as a RNA-binding protein (RBP) to bind with PAX3 mRNA. Notably, Pax3 has been implicated to regulate TGF-β/Smad signaling [13]. In this study, we speculated that FUS acted as a RBP to regulate PAX3 expression, thereby mediating AngII-induced atrial fibrosis via TGF-β1/Smad pathway, and traditional experimental techniques of molecular biology were utilized to validate this speculation.

## Materials and methods

### Plasma collection

Patients who underwent heart valve replacement surgery in The People’s Hospital of Dazu District were enrolled in this study and subdivided into the control group (patients with sinus rhythm, n = 30) and the atrial fibrillation (AF) group (n = 30). Patients with chronic pulmonary heart disease, previous coronary atherosclerotic heart disease, infective endocarditis, hyperthyroidism, severe dysfunction of liver or kidney, and malignant tumors were excluded. Their general clinical data were shown in Table 1 and their blood samples from the ulnar vein were collected and stored at −80°C after centrifugation (4,000 x g, 4°C, 15 min). The protocol was approved by the Ethics Committee of the The People’s Hospital of Dazu District and informed written consent was obtained from all participants.

**Table 1.** General clinical data of the patients in the two groups (mean ± SD)

Parameter	Control (n = 30)	AF (n = 30)
Age	55.1 ± 11.2	59.3 ± 12.8
Male/female	13/17	14/16
Cardiac function (NYHA) classification	I–II: n = 3	I–II: n = 5
	III: n = 24	III: n = 21
	IV: n = 3	IV: n = 4
SBP (mm Hg)	120.2 ± 11.5	117.7 ± 12.5
DBP (mm Hg)	82.4 ± 7.4	76.8 ± 10.3
LAD (mm)	31.9 ± 3.1	44.2 ± 3.8
RAD (mm)	37.7 ± 4	42.3 ± 3.3
LVEF (%)	53.9 ± 4.1	47.4 ± 3.6

AF, atrial fibrillation; SBP, systolic blood pressure; DBP, diastolic blood pressure; LAD, left atrium diameter; RAD, right atrial diameter; LVEF, left ventricular ejection fraction.

### Cell culture and treatment

Mouse cardiac fibroblasts were purchased from Ginio Biotechnology Co., Ltd. (Guangzhou, China) and maintained in DMEM supplemented with 10% of fetal bovine serum (FBS, Wisent Biotechnology). For creating a cellular model of AF, 1 μM AngII (MedChemExpress, Beijing, China) was utilized to incubate mouse cardiac fibroblasts for 12 h.

### Cell transfection

The short hairpin RNA (shRNA) against Fus (sh-Fus-1 and sh-Fus-2) and scramble negative control vector sh-NC, the pcDNA3.1-Pax3 and empty pcDNA3.1 vector were designed and synthesized by GenePharma (Shanghai, China). For in vitro transfection, cells were transfected with the above vectors using Lipofectamine 2000 (Invitrogen, USA) according to the manufacturer’s protocol.

### Cell counting kit-8 (CCK-8)

For cell viability assessment, normal or transfected cells were seeded onto 96-well plates with density of 2 × 10^4^ cells per well; then, subsequently exposed to 1 μM AngII for 12 h. Thereafter, 10 μL CCK-8 (Beyotime Biotechnology Co., LTD, Shanghai, China) solution was added to each well and incubated at 37°C for 2 h, and OD values were measured at 450 nm through a microplate reader (Bio-Rad Model 550, USA).

### Wound healing assay

Normal or transfected mouse cardiac fibroblasts (2 × 10^5^ cells/well) were seeded in 6-well plates and treated with 1 μM AngII for 12 h. Subsequently, 10 μL tip was used to scratch the cells. Images of the scratches were captured at 0 h and 48 h after scratch to observe cell conﬂuence. The average widths of the scratch wounds were analyzed using Image J software. The migrated distances (%) were calculated as dividing the difference between the width of the scratch at 0 h and 48 h by the width of the scratch at 0 h x100%.

### RT-qPCR

Total RNA was extracted from plasma and cells using the RNA extraction kit (Takara, China). A total of 5 μg RNA was then subjected to TaqMan one-step reverse transcription (Applied Biosystems, USA), followed by an ABI Prism 7500 sequence detector (Applied Biosystems). The specific primers for Fus and Pax3 were as follows:

Fus, forward, 5ʹ-TCAATAAATTTGGTGGTCCTCGG-3ʹ, reverse, 5ʹ-TCGGCGGGTAGCAAATGAAA-3ʹ;

Pax3, forward, 5ʹ-GCCTCAGACCGACTATGCTC-3ʹ, reverse, 5ʹ-AGATAATGAAAGGCACTTTGTCCA-3ʹ;

GAPDH, forward, 5′-AAATCGTGCGTGACATCAAAGA-3′, reverse, 5′-GGCCATCTCCTGCTCGAA-3′. Results were normalized to GAPDH expression, and a comparative CT method (2^−ΔΔCt^) was used to calculate the relative changes in gene expression.

### Western blot assay

Cells were lysed with lysis buffer (Beyotime Biotechnology) on ice for 30 min. Equal amounts (10 μg) of soluble protein were separated by 12% SDS-PAGE and transferred onto PVDF membranes (Milipore), which were then blocked with 5% nonfat milk, followed with primary antibody incubation overnight at 4°C. Secondary antibodies conjugated with horseradish peroxidase (HRP-conjugated goat anti-rabbit IgG) were used. Proteins were detected by chemiluminescence (GE Healthcare, UK). Antibodies used include (Abcam): Fus (1:5000), α-smooth muscle actin (α-SMA; 1:5000), Collagen 1 (1:1000), Fibronectin (1:1000), TGF-β1 (1:1000), phosphorylated (p)-Smad2 (1:1000), Smad2 (1:1000), p-Smad3 (1:2000), Smad3 (1:5000), Pax3 (1:1000), and GAPDH (1:5000).

### RNA-Binding Protein Immunoprecipitation (RIP)

RIP assay was conducted by Magna RIP™ RIP Kit (Millipore, USA) according to the manufacturer’s instructions. Brieﬂy, cells were lysed by RIPA lysis (Beyotime Biotechnology) and then incubated with anti-Fus antibody or IgG antibody at 4°C for 24 h. The Pax3 in immunoprecipitation complexes was detected by RT-qPCR. The IgG was used as a negative control.

### RNA pull down

RNA pull-down assay was carried out using Pierce™ Magnetic RNA-Protein Pull-Down Kit (Thermo Fisher, USA). Briefly, cells lysed by standard lysis buffer. Pax3 was labeled with biotin using Pierce RNA 3ʹ End Desthiobiotinylation Kit (Thermo Fisher, USA), then bound to streptavidin magnetic beads and incubated with cell lysates at 4°C for 1 h. The Fus protein in the RNA-protein complexes was analyzed by western blot.

### Statistical analysis

The analysis in this study was performed in GraphPad Prism 8 (GraphPad Software, CA, USA). All experiments were performed at least three times. All data were shown as the mean ± SD, with statistical differences were calculated by Student’s t-test or one-way ANOVA followed by Tukey’s test after being confirmed to be parametric and conform to normal distribution by Shapiro-Wilk test. Data with *P* < 0.05 were considered statistically significant.

## Results

### Fus is up-regulated in plasma of AF patients and AngII-induced cardiac fibroblasts

**Figure 1.**
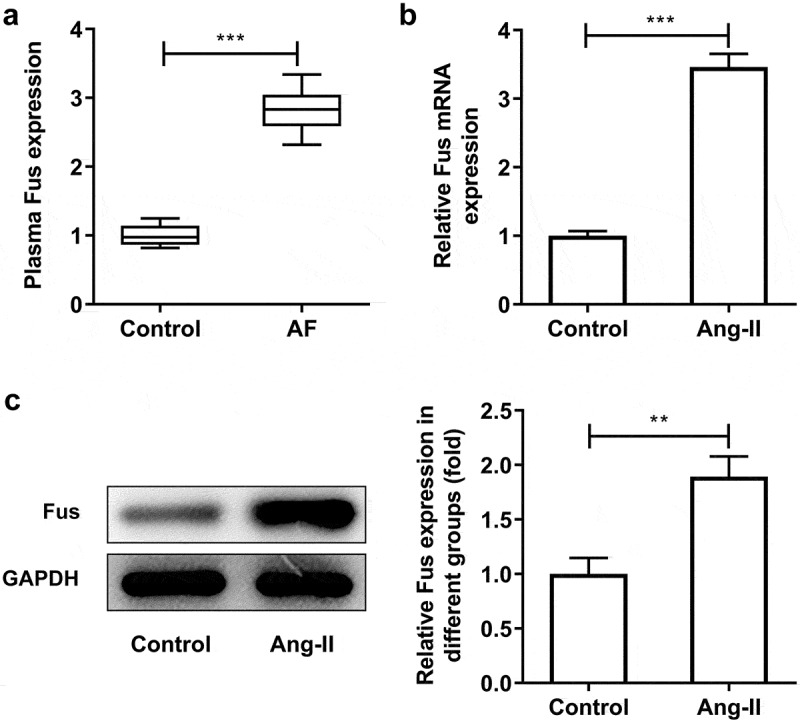
Fus is up-regulated in plasma of AF patients and AngII-induced cardiac fibroblasts. (a), the expression of FUS in plasma of patients with sinus rhythm (control, n = 30) and atrial fibrillation (AF, n = 30) was detected by RT-qPCR. (b and c), the mRNA (b) and protein (c) expressions of Fus in control mouse cardiac fibroblasts and cells that stimulated with 1 μM AngII for 12 h. **P < 0.01 and ***P < 0.001

To determine whether FUS played a role in AF, FUS expression level in plasma of control and AF patients was measured by RT-qPCR. As shown in Figure 1a, FUS was significantly up-regulated in AF group, indicating its potential role in AF. Moreover, compared with control cells, AngII-treated cardiac fibroblasts exhibited higher expression of Fus, suggesting the involvement of Fus in cardiac fibrosis (Figure 1b and c).

### Fus knockdown inhibits AngII-induced proliferation, migration, collagen synthesis and TGF-β1/Smad pathway activation of cardiac fibroblasts

**Figure 2.**
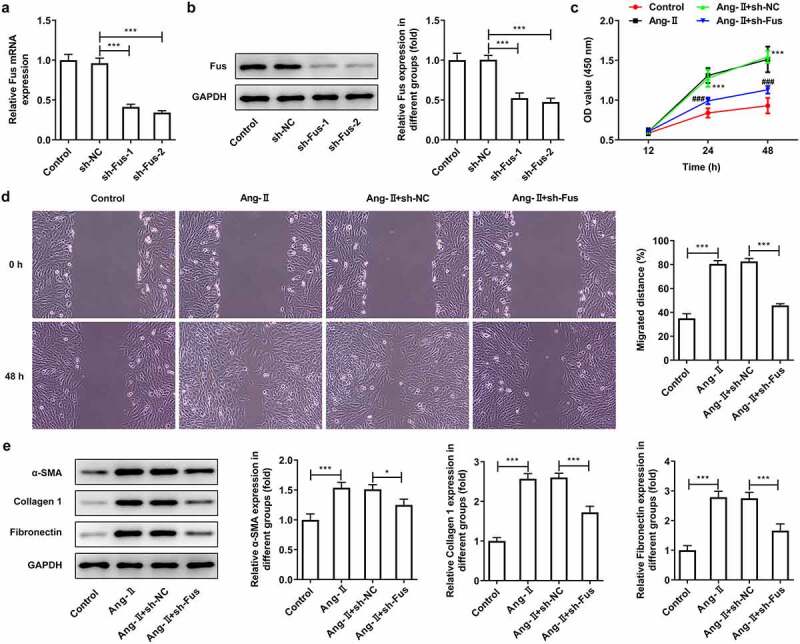
Fus knockdown inhibits AngII-induced proliferation, migration and collagen synthesis of cardiac fibroblasts. (a and b), the mRNA (a) and protein (b) expressions of Fus in control mouse cardiac fibroblasts and cells that transfected with indicated shRNAs. ***P < 0.001. (c–e), mouse cardiac fibroblasts that silenced with Fus or not were stimulated with 1 μM AngII for 12 h, then (c) the cell viability was measured by CCK-8 assay, ***P < 0.001 vs Control, ^###^P < 0.001 vs AngII + sh-NC; (d) cell migration was detected by wound healing assay; (e) the protein expression of α-SMA, collagen 1 and fibronectin was assessed by western blot. *P < 0.05 and ***P < 0.001

**Figure 3.**
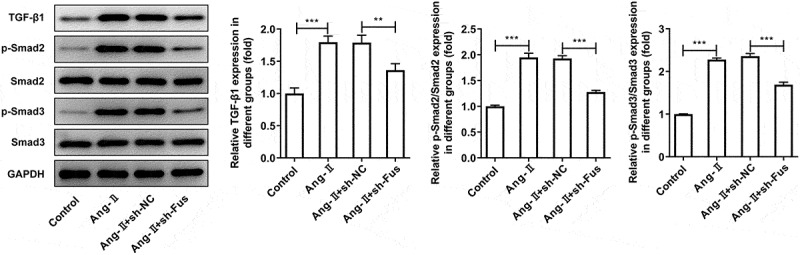
Fus knockdown inhibits AngII-induced TGF-β1/Smad pathway activation. Mouse cardiac fibroblasts that silenced with Fus or not were stimulated with 1 μM AngII for 12 h, then the protein expression of TGF-β1, phosphorylated (p)-Smad2/Smad2 and p-Smad3/Smad3 was assessed by western blot. **P < 0.01 and ***P < 0.001

Subsequently, we silenced Fus expression in cardiac fibroblasts using shRNA-Fus-1/2. The knockdown efficiency was validated using RT-qPCR and western blot assays (Figure 2a and b), and shRNA-Fus-2 was chosen for next experiments based on its better transfection efficiency. Figure 2c demonstrated that the cell proliferation was enhanced upon AngII stimulation, but Fus knockdown inhibited AngII-enhanced cell proliferation. AngII also increased the migrated distance of cells, which was instead decreased by Fus knockdown (Figure 2d). Besides, the expression of proteins involved in collagen synthesis including α-SMA, collagen 1 and fibronectin was markedly elevated in AngII-induced cells. However, this proteins expression was reduced in cells that silenced with Fus, when compared with cells that transfection with sh-NC in the presence of AngII (Figure 2e). As illustrated in Figure 3, cells that underwent AngII stimulation exerted higher expression of TGF-β1, p-Smad2/Smad2 and p-Smad3/Smad3 compared with control cells. Whereas, the expression of these proteins was down-regulated in Fus knockdown cells, compared with that in sh-NC group.

### Pax3 overexpression blocks the effect of Fus knockdown on AngII-induced TGF-β1/Smad pathway activation, proliferation, migration and collagen synthesis of cardiac fibroblasts

**Figure 4.**
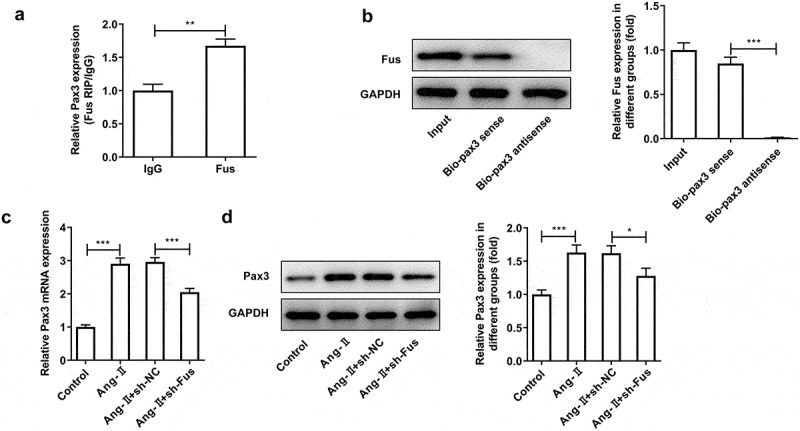
Fus binds to Pax3 and regulates Pax3 expression. (a and b), the interaction between Fus and Pax3 mRNA in mouse cardiac fibroblasts was verified by RIP (a) and RNA pull down (b) assays. (c and d), mouse cardiac fibroblasts that silenced with Fus or not were stimulated with 1 μM AngII for 12 h, then the mRNA (c) and protein (d) expressions of Pax3 were measured by RT-qPCR and western blot. *P < 0.05, **P < 0.01 and ***P < 0.001

To verify the interaction between Fus and Pax3, RIP and RNA pull down assays were performed. Results from Figure 4a and b showed that Fus could bind to the mRNA of Pax3. Meanwhile, both Pax3 mRNA and protein expression was increased upon AngII treatment, but reversely decreased after Fus knockdown (Figure 4c and d). These results indicated that, in response to AngII stimulation, Fus3 may be upregulated then to increase Pax3 expression via serving as a RBP of Pax3.

**Figure 5.**
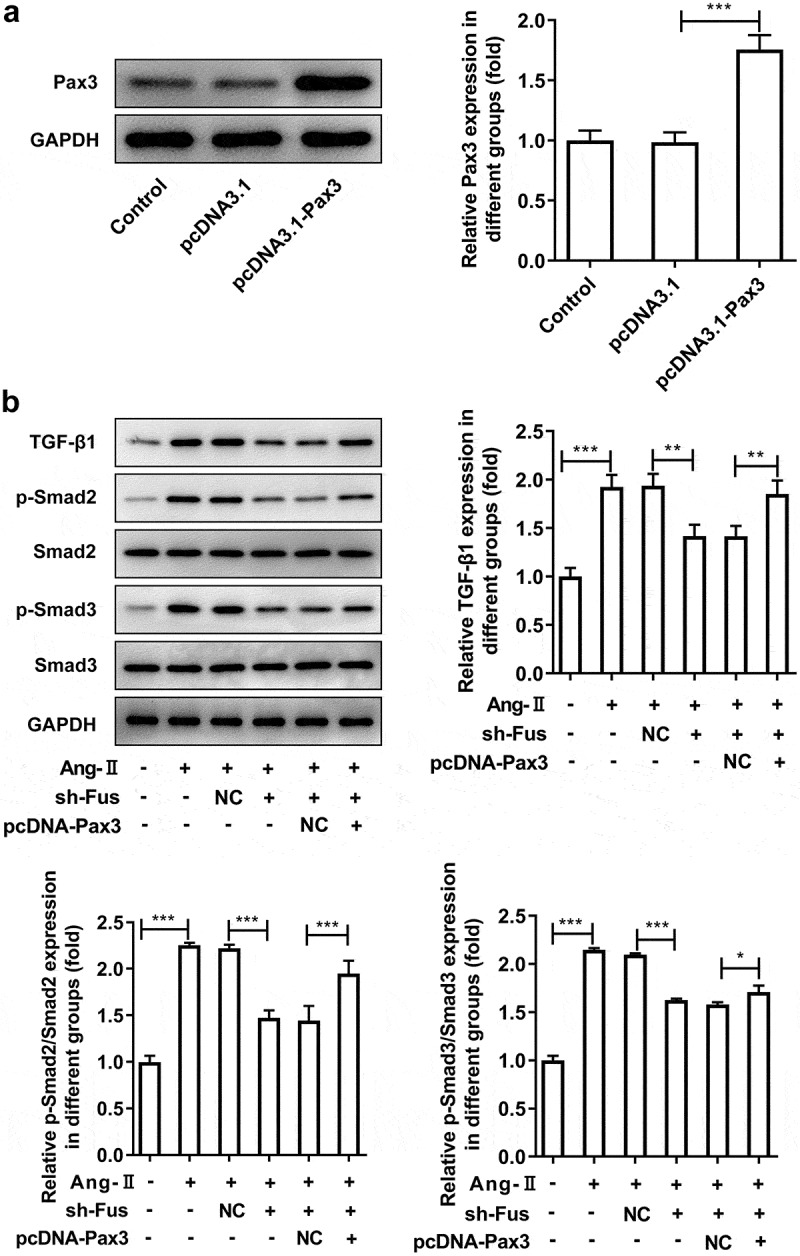
Pax3 overexpression blocks the effect of Fus knockdown on AngII-induced TGF-β1/Smad pathway activation. (a), the protein expression of Pax3 in mouse cardiac fibroblasts before and after Pax3 overexpression. (b), mouse cardiac fibroblasts that co-transfected with shRNA-Fus and pcDNA-Pax3 or not were stimulated with 1 μM AngII for 12 h, then the protein expression of TGF-β1, phosphorylated (p)-Smad2/Smad2 and p-Smad3/Smad3 was assessed by western blot. *P < 0.05, **P < 0.01 and ***P < 0.001

**Figure 6.**
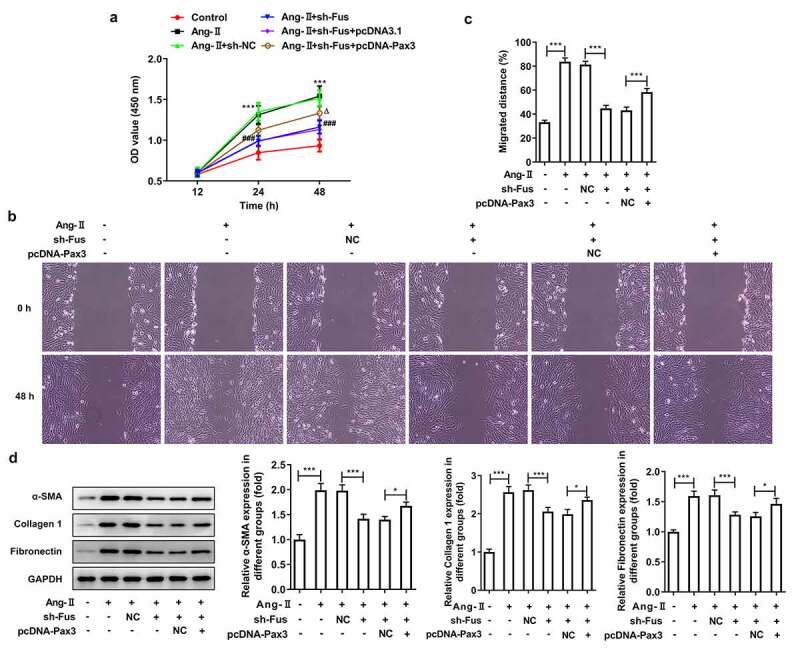
Pax3 overexpression blocks the effect of Fus knockdown on AngII-induced proliferation, migration and collagen synthesis of cardiac fibroblasts. Mouse cardiac fibroblasts that co-transfected with shRNA-Fus and pcDNA-Pax3 or not were stimulated with 1 μM AngII for 12 h, then (a) the cell viability was measured by CCK-8 assay, ***P < 0.001 vs Control, ^###^P < 0.001 vs AngII + sh-NC, ^Δ^P<0.05 vs AngII + sh-Fus + pcDNA3.1; (b and c) cell migration was detected by wound healing assay; (d) the protein expression of α-SMA, collagen 1 and fibronectin was assessed by western blot. *P < 0.05 and ***P < 0.001

Finally, to investigate whether Fus3 exerted its effect on AngII-induced cardiac fibroblasts via targeting Pax3, Pax3 was overexpressed in Fus-silenced cells, then the above experiments were repeated. The successful overexpression of Pax3 was verified by western blot assay (Figure 5a). Figure 5b showed that the protein expression of TGF-β1, p-Smad2/Smad2 and p-Smad3/Smad3 was markedly recovered in the presence of pcDNA-Pax3, compared with that in pcDNA-NC group. As shown in Figure 6a, Fus silence reduced AngII-increased cell proliferation, but this effect was blocked by Pax3 overexpression. Consistently, the cell migration was enhanced by Pax3 overexpression compared with that in cells silenced with Fus (Figure 6b and c). The AngII-increased expression of α-SMA, collagen 1 and fibronectin was reduced by Fus knockdown, but was partially recovered by Pax3 overexpression. The above data illustrated that Pax3 overexpression could inhibited all the effects of Fus knockdown on AngII-induced cardiac fibroblasts.

## Discussion

Atrial fibrosis is considered to be the common pathological basis for many causes of AF, and Fus was reported to be involved in multiple cardiac diseases [11]. In this study, we intended to investigate whether Fus could regulate atrial fibrosis thereby playing a role in AF. We found that Fus was upregulated in AF and AngII-induced cardiac fibroblasts. Moreover, Fus knockdown inhibited AngII-induced cardiac fibroblasts proliferation, migration and collagen synthesis. Mechanically, we showed that Fus could bind to Pax3 to regulate TGF-β1/Smad pathway, ultimately mediating the fibrosis of cardiac fibroblasts.

In recent years, Fus aroused concerns regarding its involvement in diverse cardiovascular diseases, such as heart failure [11], cardiac hypertrophy [12] and myocardial infarction [14]. Nevertheless, the role and specific mechanism through which Fus regulates atrial fibrosis in patients with AF remains unclear. In our study, we found that patients with AF show significantly higher Fus expression compared with patients with normal sinus rhythm. Meanwhile, Fus expression was also up-regulated in AngII-induced cardiac fibroblasts. Additionally, silence of Fus strongly prevented Ang-II activated the canonical TGF-β1/Smad pathway, thus ameliorating the profibrogenic function of cardiac fibroblasts induced by AngII in vitro. Furthermore, Fus could bind to Pax3, the overexpression of which blocked the inhibitory effect of Fus knockdown on cardiac fibrosis.

Atrial fibrosis is generally considered to be the most remarkable type of structural remodeling in patients with AF, and it is also the structural basis for the persistence of AF [3,4]. AngII is a potent vasoconstrictor, has been widely used to induce the profibrogenic function of cardiac fibroblasts both in vitro and in vivo [15]. Cardiac fibroblasts are the major contributory cells of cardiac fibrosis. In response to pro-fibrotic cytokines, cardiac fibroblasts become activated, express α-SMA and increase production of extracellular matrix (ECM) proteins such as collagens and fibronectin to form scar tissue [15,16]. In this study, our results showed that AngII promote cell proliferation and migration of cardiac fibroblasts. AngII also stimulate α-SMA, collagen 1 and fibronectin synthesis, confirming the profibrogenic effect of AngII on cardiac fibroblasts.

In our present study, RT-qPCR analysis revealed higher expression of Fus in plasma of AF patients compared with the control group. Besides, AngII resulted in an increased expression Fus in cardiac fibroblasts. Thus, we speculated that the up-regulated expression of Fus may be associated with atrial fibrosis and can serve as a biomarker in AF patients. We then silenced Fus in AngII-induced cardiac fibroblast and found that AngII-induced increased in proliferation, migration and expression in α-SMA, collagen 1 and fibronectin was effectively reversed by Fus silence. Pathological changes related to AF are mainly caused by abnormal collagen metabolism, unbalanced amounts of collagen proteins, increased collagen deposition and fibrotic diseases [17]. Our results demonstrated the inhibitory effect of Fus knockdown on AngII-induced fibrosis in cardiac fibroblasts.

Moreover, we showed that the enhanced expression of TGF-β1, p-Samd2 and p-Smad3 caused by AngII was markedly blocked by Fus knockdown. TGF-β signaling has been shown to induce fibroblast transition into activated myofibroblasts. TGF-β1 directly activates Smad signaling which triggers pro-fibrotic gene overexpression [18]. The dysregulation of TGF-β1/Smad pathway was an important pathogenic mechanism in tissue fibrosis and Smad2 and Smad3 are the two major downstream regulator that promote TGF-β1-mediated tissue fibrosis [19]. Therefore, our results indicated that Fus played its role in the profibrogenic function of cardiac fibroblasts induced by AngII via regulating TGF-β1/Smad pathway.

Fus has been reported to serve as a RBP to regulate the expression of target genes [14]. Consequently, we searched for targets of Fus in Starbase databases and identified Pax3 as a potential target gene of Fus. This finding was confirmed by RIP and RNA pull down assays. In addition, our RT-qPCR and western blot results revealed Pax3 expression was up-regulated upon AngII stimulation but instead down-regulated after Fus knockdown in mouse cardiac fibroblasts.

As a member of the PAX transcription factor family, Pax3 functions as a regulator of myogenesis [20]. Pax3 also was one of the representative target genes of β-catenin, which is involved in myofibroblast activation [21]. Furthermore, Pax3 was reported to regulate TGF-β pathway [13,22,23]. We speculated that Fus may exert its effect on cardiac fibrosis during AF via targeting Pax3. We co-transfected the pcDNA-Pax3 and shRNA-Fus into Ang-II-stimulated cardiac fibroblasts and found that ectopic expression of Pax3 reversed the influence of Fus knockdown on cardiac-fibroblast proliferation, migration, and collagen production along with TGF-β1/Smad signaling activation. These results indicated that the effects of Fus on cardiac fibrosis are mainly mediated by the up-regulation of Pax3, which in turn activated TGF-β1/Smad signaling. However, to further validate our findings, the expression level of Fus and Pax3 in heart tissues from AF patients and control donors need to be evaluated in our subsequent research. Besides, adult primary mouse fibroblasts and animal models will be constructed to investigate the effect of Fus on AF in vivo. In addition, Pax3 overexpression didn’t block the effect of Fus knockdown on AngII-induced cardiac fibroblasts totally, indicating the presence of other targets of Fus that play a role in this process, which will be explored in the future research.

## Conclusion

Taken together, we demonstrated that knockdown of Fus prevented atrial fibrosis by inhibiting cardiac fibroblast proliferation, migration, and collagen production through targeting Pax3 mRNA to inactivate TGF-β1/Smad signaling in vitro. This study for the first time illustrated the role of Fus/Pax3 axis in cardiac fibrosis. Fus/Pax3 axis may serve as a novel therapeutic or prophylactic target in AF.

## Highlights

- Fus is up-regulated in plasma of AF patients and AngII-induced cardiac fibroblasts.
- Fus knockdown inhibits AngII-induced proliferation, migration, collagen synthesis and TGF-β1/Smad pathway activation of cardiac fibroblasts.
- Fus exerts its effect on atrial fibrosis via acting as an RNA-Binding protein to regulate Pax3 expression, thus mediating TGF-β1/Smad pathway.
